# Modified vascularized interpositional periosteal connective tissue graft versus xenogeneic collagen matrix for soft tissue augmentation around implant in esthetic zone (A comparative Study)

**DOI:** 10.1186/s12903-025-07345-9

**Published:** 2025-12-12

**Authors:** Heba Ahmed Abdelmaged, Ahmed Abdelmeguid Moustafa, Ahmed Abdallah Khalil

**Affiliations:** 1 Department of periodontology, Faculty of dentistry, Minia University, Minia, Egypt; 2 Oral medicine, oral diagnosis, and Periodontology Department, Faculty of dentistry, Minia University, Minia, Egypt

**Keywords:** Immediate implant, Xenograft, MVIP-CTG, XCM

## Abstract

**Background:**

Immediate implant placement in the esthetic zone presents challenges in achieving optimal peri-implant soft tissue outcomes. Enhancing keratinized tissue thickness is critical to achieving long-term esthetic stability. The modified vascularized interpositional periosteal connective tissue graft (mVIP-CTG) has been proposed as an autogenous pedicled technique for improving keratinized tissue thickness. Xenogeneic collagen matrix (XCM) represents a less invasive alternative with reduced patient morbidity. This study aimed to evaluate and compare the clinical effectiveness of mVIP-CTG versus XCM in augmenting peri-implant soft tissue in the esthetic zone.

**Objectives:**

The primary objective was to assess and compare the effect of mVIP-CTG versus XCM on keratinized tissue thickness (KTT). Secondary objectives included evaluating changes in keratinized tissue width (KTW), pink esthetic score (PES), and radiographic buccal cortex thickness (BCT) around immediate implants over a 6-month follow-up period.

**Methods:**

This parallel-arm, assessor-blinded, randomized controlled trial included twenty patients requiring the extraction of a hopeless single anterior tooth. Patients were randomly allocated into two equal groups. One group received immediate implant placement, xenogeneic bone grafting, and mVIP-CTG, while the other groupreceived immediate implant placement, xenogeneic bone grafting, and XCM.Cone-beam computed tomography (CBCT) scans were obtained at baseline (prior to extraction) and at 6 months postoperatively. Clinical parameters, including KTT, KTW, and PES, were assessed at baseline, 3 months, and 6 months. BCT was measured radiographically at baseline and 6 months using CBCT. Statistical analysis was performed using IBM SPSS® version 26. Data normality was evaluated using the Shapiro–Wilk test. Independent and paired t-tests were used for intra- and intergroup comparisons—repeated measures ANOVA was employed to assess changes over time. A *p*-value < 0.05 was considered statistically significant.

Ethical approval was obtained from the Faculty Research Ethics Committee, Minia University (543/84/2021). The trial was retrospectively registered on ClinicalTrials.gov (NCT06808243) on February 5, 2025.

**Results:**

All patients completed the 6-month follow-up without dropout. There were no statistically significant differences between groups regarding KTW, PES, or BCT. However, the mVIP-CTG group demonstrated a significantly greater increase in KTT compared to the XCM group (*p* < 0.05).

**Conclusion:**

The pedicled mVIP-CTG technique resulted in a superior increase in keratinized tissue thickness around immediate implants compared to XCM. Despite this, XCM offered advantages in terms of reduced morbidity and shorter surgical time, suggesting it remains a viable alternative in cases where patient preference or clinical limitations preclude autogenous grafting.

## Introduction

Maintaining the soft tissue volume and contour in harmony with the adjacent soft tissues of surrounding teeth is seen as crucial for preserving the natural aesthetic appearance, particularly in the aesthetic zone. Delaying implant placement until the complete healing of hard and soft tissues often requires supplementary surgical procedures, such as guided bone regeneration or ridge splitting, or leads to implant placement in less-than-ideal positions, resulting in unsatisfactory aesthetic outcomes to counteract alveolar ridge resorption [1].

Immediate implant placement (IIP) in the esthetic zone is one approach for replacing a hopeless anterior tooth that can provide predictable and esthetically pleasing outcomes [2]. For proper positioning of the implant, a minimum of 2 mm palatal from the inner aspect of the buccal socket wall helps alleviate the extent of bone resorption following tooth extraction. It is advisable to graft the space between the socket and the implant, which is called the jumping gap [3]. The objective of the grafting technique is to fortify the hard tissue encircling the implant. This treatment is thought to affect the soft tissue surrounding the implant beneficially. Strengthen. A crucial component influencing the shape of soft tissue is buccal cortex thickness (BCT). Variations in BCT are regarded as a significant predictor of the aesthetic outcome of the immediate implant, evaluated by the Pink Esthetic Score (PES) [4].

Despite the concurrent application of guided bone regeneration to rectify peri-implant bone defects or to fill horizontal gaps between implants and socket walls, alterations in soft tissue levels may still transpire, especially in cases of slender gingival biotypes or insufficient keratinized tissue width [5]. To mitigate the risk of mucosal recession, sustain a consistent peri-implant mucosal level, and address alterations to the peri-implant mucosal layer following “Immediate Implant Placement and Restoration” (IIPR), it is advisable to enhance the soft tissue and augment the volume of keratinized tissue utilizing autogenous soft tissue grafts or tissue substitutes [6].

To improve wound closure and increase the width of keratinized tissue (KTW), Edel initially suggested using autogenous soft tissue grafts after implant placement, which is still the gold standard for conserving and increasing peri-implant tissue [7, 8].

One method for pedicle CT grafting is the VIP-CT flap. The VIP-CT approach effectively facilitates the concurrent development of hard and soft tissue at the implant site, enabling the repair of significant cosmetic ridge abnormalities [9]. The incisive papilla, essential for positioning the harvested graft, was pivotal in the advancement of this procedure, which considers the neurovascular supply of the premaxilla and palate. The VIP-CT flap addresses multiple limitations of prior procedures. These factors encompassed graft length, color matching, vascularity, postsurgical contraction, limited thickness, and prolonged operating time [10].

In 2012, Kim et al. presented a modified vascularized interpositional connective tissue (mVIP-CT) graft, incorporating a papilla preservation flap at the implant site to mitigate postoperative flap shrinking and avert papillary loss. This alteration of the VIP-CT flap, together with palatal tunneling, decreases tissue damage, hence decreasing postoperative shrinkage and improving the vascular integrity of the donor site [11]. Various items have been utilized as alternatives to connective tissue grafts to address issues in palatal donor sites, including the xenogenic collagen matrix (XCM) [12].

XCM is an innovative absorbable bio-membrane derived from pigs, designed to overcome the limitations of autologous tissue grafts. The dermis of pigs is the origin of type I and type III collagen. The new connective tissue entirely supplants the old matrix after approximately 6 to 9 months, functioning as a scaffold for the infiltration of blood vessels and cells [13].

This clinical trial sought to compare mVIP-CTG with XCM after three and six months to assess soft tissue alterations in the cosmetic zone surrounding immediate dental implants. Furthermore, after the 6-month follow-up period, assess the radiographic alterations in BCT.

## Materials and methods

### Study design

**Fig. 1:**
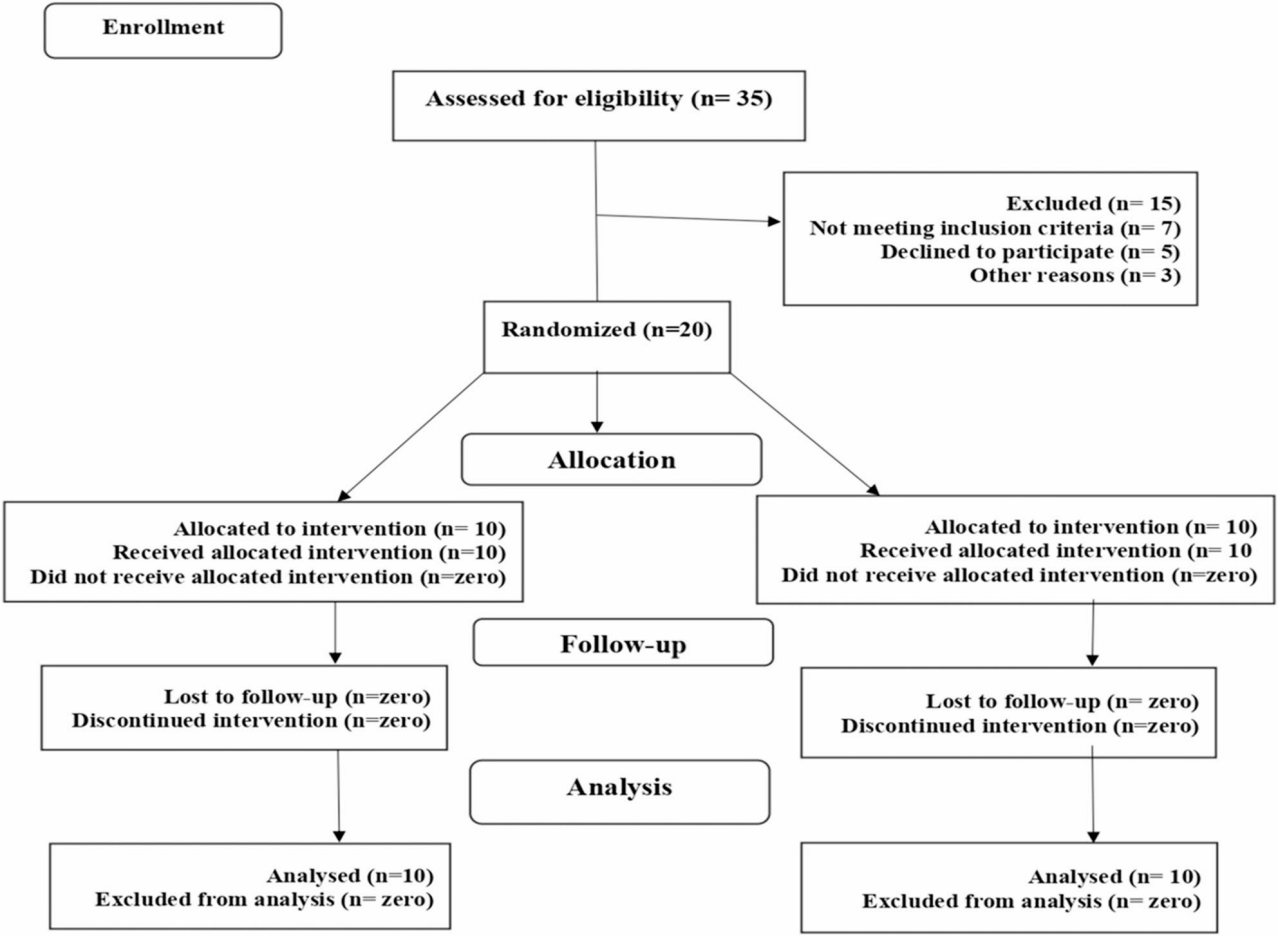
CONSORT flowchart showing participant allocation, follow-up, and analysis

This study was retrospectively registered as a clinical trial in https://clinicaltrials.gov/on02/5/2025 under registration number (NCT06808243). Following the CONSORT standard for clinical trials, the research was intended to be a clinical trial that was conducted in parallel, with a single blind design as only the outcome assessors and the statistician were blinded. It was controlled by randomization (Fig. 1). All patients received immediate implants in the esthetic zone. Patients were randomly allocated into two equal groups: the mVIP-CTG group and the XCM group.

#### Patient selection

 A total of twenty patients, comprising fourteen males and six females, were meticulously selected from the outpatient clinic of the Periodontal Department at Minia University, Faculty of Dentistry, Egypt. Patients received comprehensive information on the procedure, the type of prosthesis, and any associated hazards—all individuals who provided consent signed written informed consents. The Faculty Ethical Committee of Scientific Research accepted the study protocol (543/84/2021).

#### Sample size calculation

 The necessary quantity of implants in each cohort was established following a power analysis utilizing G Power 3.1.9.2 software (University of Düsseldorf, Düsseldorf, Germany). Data derived from prior literature [14] were utilized to compute the effect size based on the thickness of keratinized tissue, which serves as the primary outcome. This resulted in a minimal sample of 20 implants (10 patients in each group) to achieve 80% power at a 5% significance level. No participants withdrew from the trial. An unaffiliated statistician conducted this analysis.

The scientists utilized a computer-generated randomization procedure to randomly assign patients to one of two groups, as detailed below: Both groups received immediate implants and xenograft; however, soft tissue augmentation was performed using mVIP-CTG in group I and XCM in group II, with each group consisting of 10 patients.

Patients were deemed eligible if they satisfied the subsequent inclusion criteria: (1) Participants must be in good health and aged between 20 and 40; (2) Participants must possess teeth that require extraction owing to trauma, crown or root fractures, severe carious lesions, or external root resorption. (3) No pathological abnormalities were seen at the implant recipient site. No probing pocket depths over 4 mm suggest periodontal health. Exclusion Criteria: Patients undergoing radiation therapy, using bisphosphonates, or who have severe osteoporosis are considered absolute contraindications. Parafunctional habits such as clenching, bruxism, smoking, intoxication, pregnancy, or lactation are considered local contraindications. Systemic illnesses that may affect osseointegration, metabolic problems, or unresolved periodontal infections are considered relative contraindications.

### Randomization and blinding

Twenty individuals were randomly allocated to either group I (the mVIP-CTG) or group II (the XCM) via a closed envelope approach for fundamental randomization. Each group received a set of 10 cards, comprising ten cards marked with the sign I and ten cards marked with the symbol II. Upon the random selection of a card, which was to be presented to a secretary not engaged in the assessment of research parameters, eligible patients were assigned to their respective groups based on the symbol on the chosen card. The statistician and clinical outcome assessors were blinded to group allocation; however, blinding the surgeon and patients was impractical due to the nature of the procedures.

#### Presurgical procedures

 All patients meeting the inclusion criteria received an initial clinical and radiographic assessment, accompanied by a preoperative CBCT to assess the underlying bone condition, select the appropriate implant length and diameter, and quantify bone height and width. All trial participants underwent phase I therapy to establish suitable oral hygiene conditions, and the patients were encouraged to utilize a chlorhexidine mouth rinse (0.12%) prior to surgery and twice daily postoperatively before commencing extraction and implant placement.

#### Surgical procedures

**Fig. 2:**
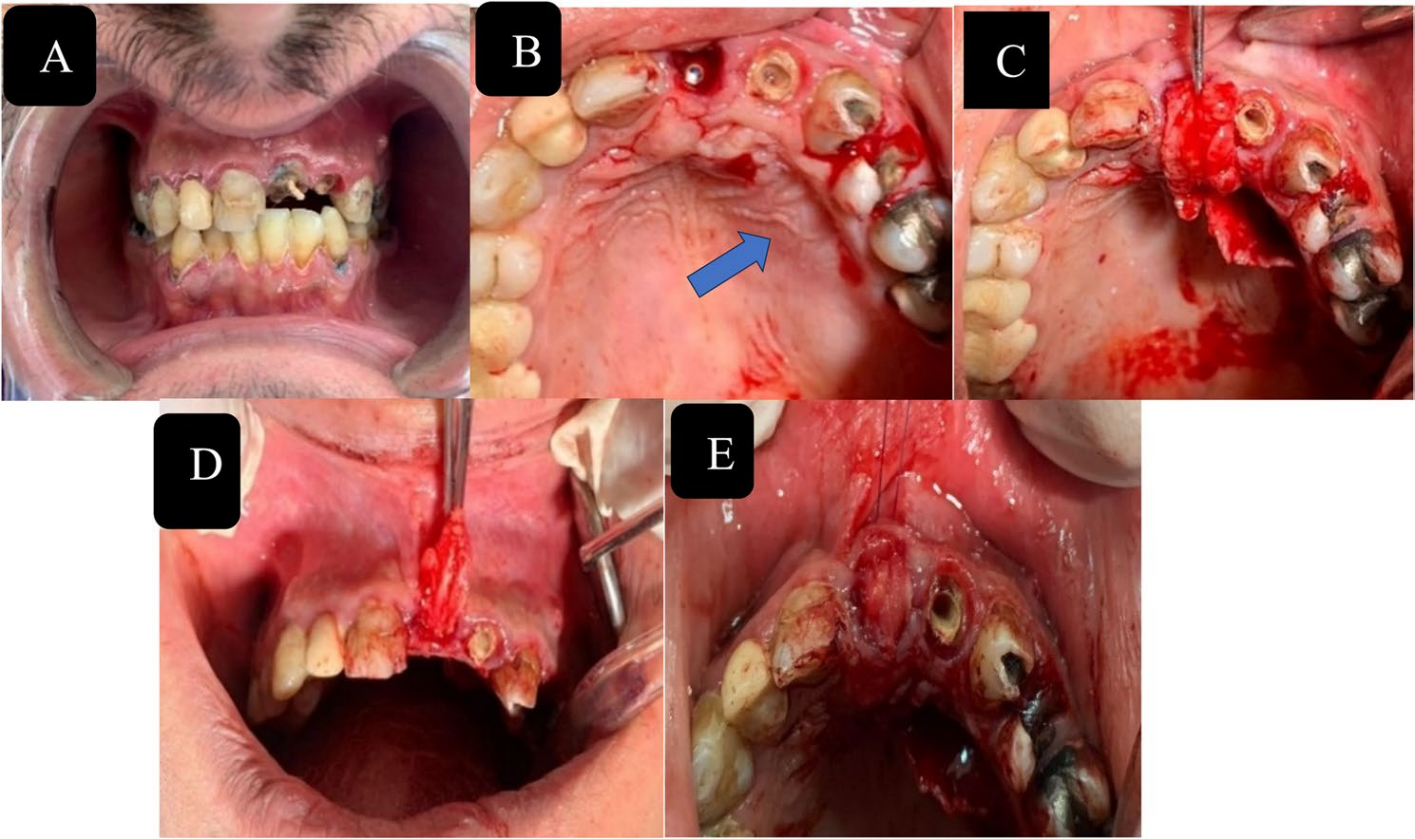
Surgical steps for group I (mVIP-CTG). **A** Facial view of an unrestorable maxillary Central incisor, (**B**) Modified single incision given for connective tissue harvest (Blue arrow), (**C**) ensure that the graft can cover the buccal plate, (**D**) Palatal tunnelling done near the recipient site for advancement of pedicle graft (**E**) anchoring the pedicle graft in buccal pouch

**Fig. 3:**
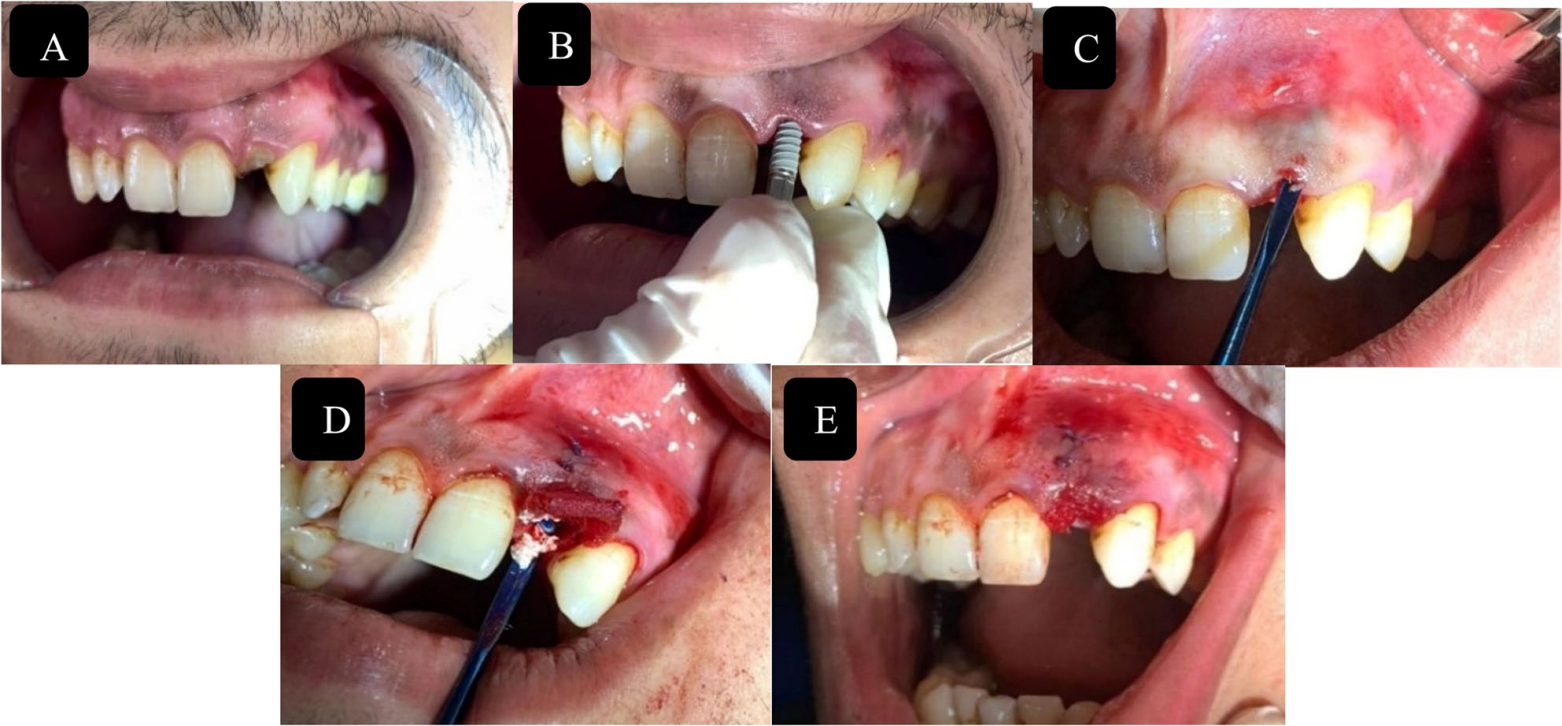
Surgical steps for group II (XCM). **A** Facial view of an unrestorable maxillary lateral incisor, (**B)** Immediate implant placement in the extraction socket, (**C**) Buccal Pouch for buccal placement of XCM (**D**) membrane stabilized buccally and bone graft placed in jumping gap (**E**) complete stabilization of the membrane with external mattress sutures over the implant

 After the application of a deep local anesthetic, a gentle extraction was conducted, followed by the immediate implantation of an implant. The accurate three-dimensional placement of the implant was confirmed, and the space between the implant and socket was filled with a xenograft (Cerabone, Botiss Biomaterials, Berlin, Germany). A modified single incision approach was employed to procure the mVIP-CT transplant from the palate in group I. This was subsequently succeeded by progression via a palatal tunneling procedure. An anteroposterior subperiosteal tunneling was performed on the palate at the implant site to establish a conduit for the pedicle graft linking the donor and recipient sites. Subsequent to the atraumatic extraction, the anterior extension of the tunnel was established beneath the margin of the extraction socket. A solitary incision was executed on the palate, and the posterior segment of the tunnel was prepared mesially to it. The tunnel’s passageway was subsequently examined with a gadget upon completion. The flap was subsequently advanced over the jumping distance via the tube to the implant site. The pedicle graft was secured in the prepared buccal pouch using external mattress sutures. At the same time, the donor site was reinforced with cross-mattress sutures utilizing 5–0 Prolene suture (Fig. 2). In group II, the XCM (Mucoderm, Botiss) was rehydrated using sterile saline solution and subsequently trimmed using scissors to conform to the size of the buccal pouch. Following the administration of local anesthesia and the execution of an atraumatic extraction, a crestal submucosal incision was made, subsequently leading to the placement of the implant. Precise dissection techniques were employed to raise a flap with a split-thickness incision. An expansion of the split-thickness flap was executed at the junction of the buccal aspect and the crest, resulting in the formation of a buccal split-thickness pouch. The dimensions of the pouch were determined by considering the buccal aspect of the implant site and the buccal surfaces of the two adjacent teeth (Fig. 3).

#### Postsurgical instructions and follow-up

 Post-surgery, patients were instructed to rinse twice daily with a 0.2% chlorhexidine solution for 5 days and to uphold excellent oral hygiene, especially in the augmented region. Furthermore, they were prescribed a 7-day regimen of antibiotics and painkillers. The extraction of gingival sutures transpired 10 days after the surgical procedure. Control assessments were performed at one day, ten days, thirty days, three months, and six months post-operation. The augmented area was clinically assessed to detect any difficulties in wound healing.

### Prosthetic protocol

In the prosthetic phase, after implantation and soft tissue augmentation, all patients in both cohorts received standardized provisional and definitive restorations. All cases utilized the identical implant technology (Oxy Dental Implant), abutment type, and prosthetic materials. A competent prosthodontist executed all restorations utilizing a standardized fabrication technique to guarantee consistency across both groups.

### Ethical consideration

The study was conducted following the Declaration of Helsinki and received approval from the Ethical Committee of Scientific Research at the Faculty of Dentistry, Minia University, under protocol number (543/84/2021).

### Measured outcomes

#### Primary Outcome

Variation in keratinized tissue thickness (KTT) from baseline to 3 and 6 months, assessed utilizing a standardized stent and endodontic file.

#### Secondary Outcomes

Keratinized tissue width (KTW), Pink Esthetic Score (PES), and buccal cortical thickness (BCT).

#### Clinical assessment

 Include the following measured parameters at baseline (immediately after IIP), 3, and 6 months.

**Fig. 4:**
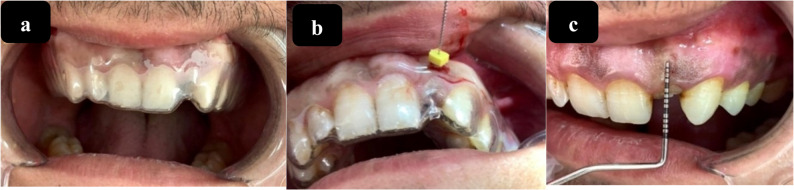
Clinical assessment procedures of the gingival tissue. **a** Customized stent for KTT measurement; (**b**) Measuring the keratinized tissue thickness using the stent and endo file; (**c**) Measuring the width of keratinized tissue using UNC15
- b- keratinized tissue width (KTW): Measured at the mid-buccal area from the gingival crest to the mucogingival junction using a UNC-15 periodontal probe (Fig. 4c).
- c- Pink esthetic score (PES): (score range: 0–14) was assessed two months after the prosthetic operation. A digital camera was employed to acquire two photos of each implant: one depicting the implant’s facial aspect and the other illustrating its occlusal region. The PES consists of seven evaluative variables: We allocated scores of 2, 1, or 0 to each of the seven PES criteria: Mesial and Distal Papilla, soft tissue level, contour, alveolar process deficits, color and texture of soft tissues, with a maximum possible score of 14 [15]. The PES was assessed by a solitary, seasoned, and calibrated clinician. Calibration was conducted before data collection with standardized clinical images and reference cases. Due to the restricted sample size, no inter-rater evaluation was performed.

- a- keratinized tissue thickness (KTT): To ensure the consistency of all measurements, a #25 endodontic K-file with a rubber stopper was utilized through an aperture in a bespoke vacuum stent created for each patient. This stent was explicitly designed to include the occlusal surfaces of the teeth next to the augmentation site. The same calibrated examiner conducted all measurements to guarantee uniformity. Gentle, constant pressure was applied during probing, and intra-examiner reliability was evaluated through repeated measurements on five randomly selected cases, resulting in an intra-class correlation coefficient (ICC) of 0.91, signifying excellent reliability [15] (Fig. 4a and b).

Buccal cortex thickness (BCT) was assessed utilizing CBCT scans acquired at baseline and six months postoperatively. Image capture and reconstruction were executed utilizing a cone beam CT system, while image superimposition was conducted with third-party software (OnDemand3D, version 1.0.10; Cybermed, Korea). The preoperative image was aligned with the postoperative image using manual registration, using cranial landmarks. The software automatically completed the registration (superimposition), ensuring optimal accuracy. Each image, both primary and secondary, was assigned a color code for identifying purposes. The initial measurements were documented on the primary image. The measurement on the primary image was retained, but the primary image itself was discarded, resulting in the secondary image remaining. A fresh measurement was obtained from the secondary image aligned with the same plane direction and cut from the main image to ensure standardization.

**Fig. 5:**
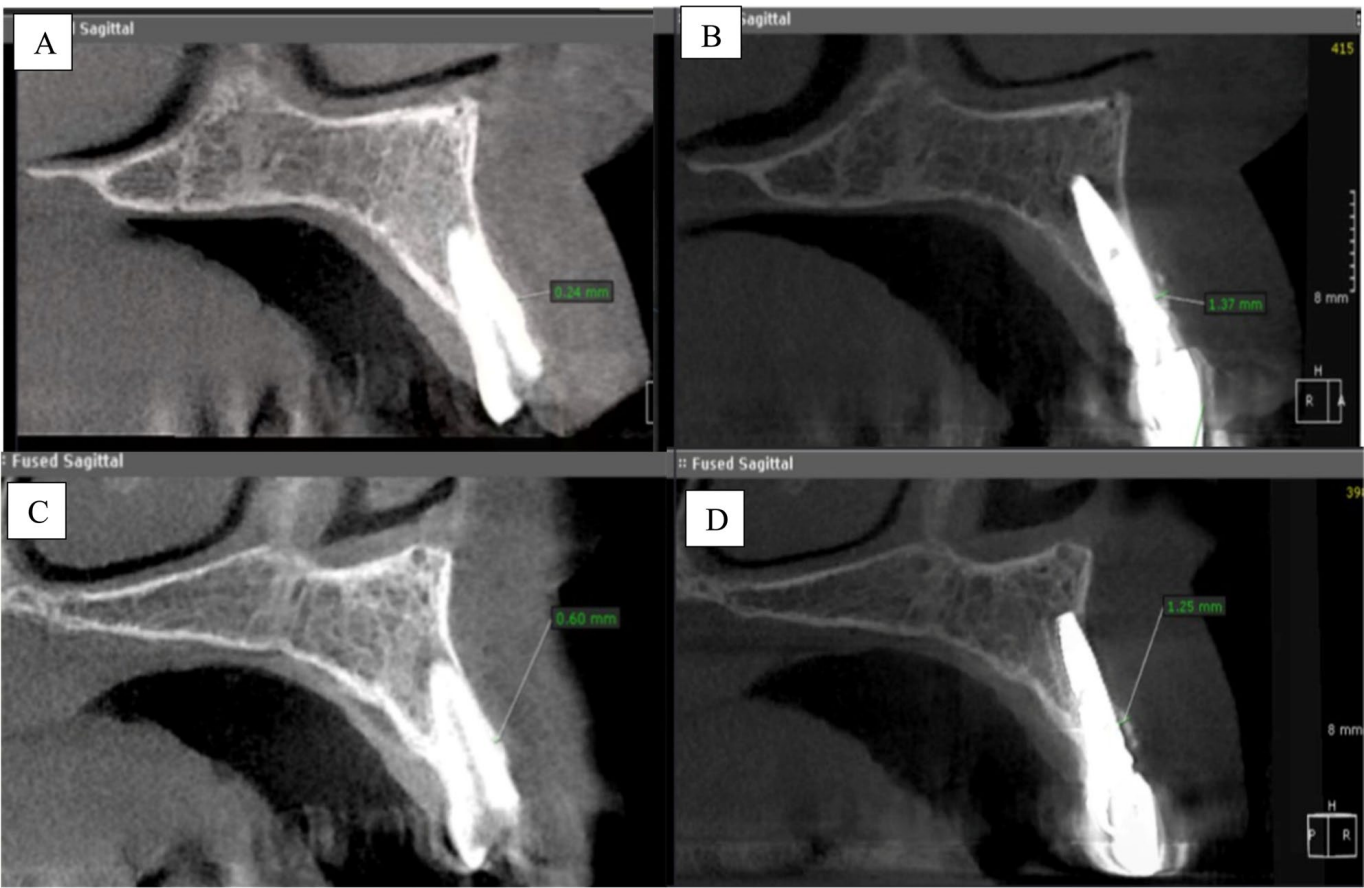
Radiographic superimposition to assess buccal cortex thickness (BCT). **A** a sagittal section revealing BCT before the surgical procedure. (**B**) a sagittal section of the superimposed radiograph revealing BCT changes after 6 months of using mVIP-CTG. (**C**) a sagittal section revealing BCT before the surgical procedure (**D**) a sagittal section of the superimposed radiograph revealing BCT changes after 6 months of using XCM

To evaluate horizontal alterations in the labial bone, the initial image was obscured, and a measurement line was delineated from the labial surface of the implant to the labial cortical plate in the secondary (postoperative) image. The identical line was subsequently stretched to the matching labial bone surface on the primary (preoperative) picture following reactivation, assuring standardization. A blinded radiologist conducted all measures, and a second radiologist replicated the measurements on a random subset of five instances. Inter-examiner reliability was assessed using the intra-class correlation coefficient (ICC), resulting in a value of 0.88, signifying substantial agreement. The alterations in BCT were illustrated by fused pre- and postoperative images [16] (Fig. 5).

### Statistical analysis

The data is analyzed by a statistician who is unaware of the research design or groupings, utilizing IBM SPSS^®^ (ver. 26, SPSS Inc., IBM Corporation, Armonk, NY, USA), and assessed for normality via the Shapiro-Wilk test on the data. The statistical data was presented utilizing the standard deviation and mean. The two groups’ means were compared using an independent t-test. A paired t-test was utilized to compare the two means within each group before and after therapy. To assess the impact of an intervention on the means of each group over time, one should employ a repeated measures ANOVA. A *p*-value below 0.05 was considered to signify a statistically significant level.

## Results

No patients withdrew or experienced implant failures among the twenty participants (14 females and six men) aged 20 to 40 years. All participants adhered to the treatment programs, and the implants achieved effective osseointegration without problems. The data is presented as mean ± standard deviation.

### Clinical parameters

#### I-Keratinized tissue thickness

- Comparison between groups

The KTT values were 2.8±0.56 mm for the mVIP-CTG group and 2.0±0.55 mm for the XCM group, respectively. A statistically significant difference was noticed between the two groups' KTT at baseline, 3 months, and 6 months (*P*-value = 0.7, *P*-value= 0.04*, and *P*-value = 0.004*, respectively), with no significant differences noted between the groups at baseline.

- Changes within each group

KTT was elevated from baseline to 3 and 6 months in both cohorts, with a more pronounced enhancement observed in the mVIP-CT group compared to the XCM group at both time points Table 1.

**Table 1:** Descriptive statistics and results of the clinical assessment (KTT, KTW, and PES) between groups

Clinical data		Groups		*P* value (95% CI)	Effect size (Cohen’s d)
		mVIP-CTG (*n* = 10) Mean ± SD	XCM (*n* = 10) Mean ± SD		
KTT	**baseline** **3 months** **6 months**	1.5 ± 0.53 1.9 ± 0.41^a^ 2.8 ± 0.56^b^	1.4 ± 0.67 1.5 ± 0.40 2.0 ± 0.55	0.7 (−0.47, 0.67) 0.04* (0.019, 0.78) 0.004* (0.28, 1.32)	0.16 0.98 1.44
KTW	**baseline** **3 months** **6 months**	1.4 ± 0.52 1.5 ± 0.53 2.0 ± 0.84	1.6 ± 0.52 1.7 ± 0.52 1.9 ± 0.68	0.3 (−0.69, 0.29) 0.4 (−0.69, 0.29) 0.7 (−0.62, 0.82)	−0.38 −0.38 0.13
Pink esthetic score (PES)		6.8 **±** 0.57	6.3 **±** 0.53	0.06 (−0.017, 1.02)	0.91

N.B. *Statistically significant difference between two groups

^a, b^Statistically significant difference across time within the group (a; at 3 months vs 6 months and b; at baseline vs 6 months)

#### II- keratinized tissue width (KTW)

No statistically significant difference was observed between the KTW scores of the two groups at baseline, three-month, and six-month follow-ups (*P*-values = 0.3, 0.4, and 0.7, respectively).

- Variations within each group

KTW scores exhibited temporal variations in both the mVIP-CTG and XCM groups. No statistically significant change was observed from baseline to 3 months, and there was a statistically nonsignificant increase in KTW scores at 6 months, based on pairwise comparisons across time intervals Table 1.

#### Pink Esthetic Score (PES)

The PES scores of the two groups exhibited a non-statistically significant difference at the six-month follow-ups (*P*-value = 0.06). The PES scores were 6.8 ± 0.57 for the mVIP-CTG group and 6.3 ± 0.53 for the XCM group, demonstrating a minor but nonsignificant increase in PES in the mVIP-CTG group compared to the XCM group.

#### Buccal cortex thickness

At baseline, there was no statistically significant difference in BCT values between the two groups (*P*-value = 0.2). After six months, a statistically significant change was noted between the groups (*P*-value = 0.06).

#### Changes within each group

The BCT scores in the mVIP-CTG group rose from 0.744 ± 0.33 at baseline to 1.207 ± 0.16 at six months, but in the XCM group, BCT had a marginal increase from 0.92 ± 0.32 mm at baseline to 0.97 ± 0.31 mm at six months Table 2.

**Table 2:** Descriptive statistics and results of radiographic assessment of BCT between groups

Radiographic data		Groups		*P* value (95%CI)	Effect size (Cohen’s d)
		mVIP-CTG (*n* = 10) Mean ± SD	XCM (*n* = 10) Mean ± SD		
Buccal cortex thickness	**Baseline** **6 months**	0.744 ± 0.33 1.207 ± 0.16	0.92 ± 0.32 0.97 ± 0.31	0.2 (−0.48, 0.13) 0.06 (0.0052, 0.47)	−0.54 0.96
	***p***- value	0.01^#^	0.6		

## Discussion

Immediate implant insertion (IIP) provides significant benefits for both doctors and patients, including a notable economic and societal impact due to the decrease in the number of procedures, treatment duration, and patient satisfaction [17].

Adequate keratinized tissue thickness guarantees an optimum biological width around the implants, establishing a barrier against pathogens and risks in the oral environment. The peri-implant soft tissue also masks any metallic shadow from the implants and abutments, improving the esthetic outcome. Employing a soft tissue graft following immediate implant placement may be beneficial in reducing the occurrence of peri-implant mucosal recession, augmenting keratinized tissue thickness, and limiting physiological tissue remodeling [18].

The use of pedicle grafts indicates a favorable prognosis, as the tissue augmentation is significant and the vascular supply derives from the connective tissue periosteal plexus within the flap, leading to straightforward postoperative recovery. Furthermore, it facilitates both vertical and horizontal soft tissue volume augmentation. The donor site is primarily safeguarded, yielding a more advantageous risk-to-benefit ratio in comparison to alternative harvesting techniques [19].

Several alternatives for thickening soft tissues have been proposed as potential answers to the issue of donor sites [20]. Various collagen matrices have been investigated as viable alternatives to connective tissue grafts in both therapeutic and preclinical contexts [21]. Acellular three-dimensional collagen matrix sourced from porcine dermis is produced by Botiss Biomaterials GmbH in Zossen, Germany, and is referred to as the xenogenic collagen matrix mucoderm^®^ (CM). The composition is organic and comprises type I and type III collagen. New connective tissue supplants the old after approximately 6 to 9 months, functioning as a framework for cellular and vascular ingrowth (the matrix) [13].

This study aimed to improve the cosmetic outcomes of implants in the aesthetic zone by comparing the clinical and radiographic analyses of mVIP-CTG with those of XCM. We examined the alterations in buccal soft and hard tissue contours after 3 and 6 months post-surgery. We employed several outcomes, including KTT, KTW, PES, and radiographic alterations in buccal bone thickness (BCT), as proposed by Levine et al., as these elements influence the aesthetic result of the implant restoration [22].

The gap in the bone was augmented with graft material to optimize outcomes. Numerous studies have demonstrated that the application of biomaterials in gap grafting enhances bone quantity following site healing and significantly reduces horizontal bone resorption in the buccal bone that occurs immediately after implantation in fresh extraction sockets [3]. Nevertheless, research indicates that the preservation of the buccal bone plate is more critical for long-term success than the presence of biomaterials [23].

Neither the XCM nor the mVIP-CTG groups exhibited inflammation or inadequate clinical integration in our study. Both groups demonstrated commendable peri-implant aesthetic results as evaluated by PES; however, the mVIP-CTG group attained marginally superior outcomes, which did not achieve statistical significance (*P* = 0.06). The elevated PES may result from the selection of cases based on periodontal health, buccal bone integrity, the number of adjacent natural teeth, and the absence of diastemas, all of which diminish the probability of aesthetic issues. The clinical investigation conducted by Cosyn et al. [24] revealed no statistically significant differences between CTG and XCM for PES, and the systematic review by Ashurko et al. [25], which contradicts our results.

Keratinized tissue thickness (KTT) constituted a primary outcome assessed in this study. Linear measurements were recorded at baseline, three months, and six months post-surgery, and the results were analyzed both within and between the two groups. The KTT markedly increased in the mVIP-CTG group from 1.5 ± 0.53 mm (baseline) to 2.8 ± 0.56 mm (after 6 months), but in the XCM group it ascended from 1.4 ± 0.6 mm (baseline) to 2.1 ± 0.6 mm (after 6 months). The mVIP-CTG group demonstrates a statistically significant difference (*P* < 0.004).

One plausible explanation for these findings is that the mVIP-CTG and XCM were placed in a split-thickness buccal pouch to enhance blood flow, a critical factor in the healing process [26]. mVIP-CTG showed a statistically significant positive impact on gingival levels in the midbuccal region. A surgical approach utilizing a pedicled palatal flap of appropriate thickness, along with the anterior palatal mucosa and labial gingival flap, may explain its minimal impact. Thus, it maintains the integrity of blood vessels and diminishes the likelihood of contracture post-surgery.

One possible explanation for the earlier degradation and subsequent volume reduction of XCM may explain the superior gain in KTT observed with CTG compared to XCM. By about day seven, autogenous tissue enhances revascularization and cellular viability at an accelerated pace. Collagen membranes need to disintegrate gradually, so enhancing the probability that newly synthesized collagen fibers will mature appropriately [27].

Numerous studies have investigated the efficacy of collagen matrices, yielding inconclusive results. For instance, Cairo et al. [28] discovered that six months after CTG use, keratinized tissue thickness increased at a significantly higher rate than XCM, and Schmitt [13] discovered that SCTG outperformed XCM; nevertheless, their results do not indicate a statistically significant difference, which contradicts our findings. According to a comprehensive review by Vallecillo et al. [29], groups utilizing SCTG showed a more significant growth in tissue compared to those employing XCM, based on 19 evaluated studies on the subject.

No significant difference was seen between the two groups (*P* value = 0.7). The mVIP-CTG group exhibited an increase in KTW from 1.4 ± 0.52 mm at baseline to 2.0 ± 0.84 mm after 6 months, whereas the XCM group increased from 1.6 ± 0.52 mm at baseline to 2.3 ± 0.6 mm after 6 months. A systematic study corroborated our findings, indicating that XCM elevated KTW rates comparable to CTG, but KTT exhibited significantly inferior outcomes relative to CTG [27].

The clinical trial of Ashurko et al. [30] corroborated our findings; it demonstrated that the two techniques nearly reached the same end amount of KTW without a discernible difference.

Our findings are at odds with those of a clinical trial [1] and a systematic review that found that groups treated with CTG in addition to immediate implant had considerably wider keratinized tissue.

Statistical analysis indicated a significant difference between the two groups from baseline ‎‎(0.744 ± 0.33) to (1.207 ± 0.16) at 6 months (*P* = 0.01), although this difference was not consistent across all time points (*P* = 0.06). Our findings indicate that the mVIP-CTG group exhibited a marginally larger enhancement in buccal cortical thickness relative to the XCM group. One potential explanation is the accelerated matrix breakdown and tissue turnover that transpires within the initial three months after keratinized tissue thickening.

Another potential reason is that the primary wound closure and compression induced by the thicker mucoderm (XCM) in comparison to pedicled CTG may be counterproductive regarding clinical efficacy. The preparation of the buccal pouch may have compromised the vascular integrity of the labial tissues, perhaps negatively impacting the healing of the buccal bone. The mVIP-CTG group has a diminished effect; however, it may have contributed to the modest increase in BCT observed in both groups.

The reduction in buccal bone thickness (− 0.84 ± 0.61 mm) was considerably greater in the connective tissue grafted group compared to the non-grafted group (− 0.46 ± 0.54 mm; *p* = 0.02), which contradicts our results [4]. Rather than safeguarding the buccal bone plate during remodeling, soft tissue augmentation may have exacerbated buccal bone resorption. The preparation of the pouch compromised the vascular integrity of the labial tissues, and the inevitable bone remodeling following extraction adversely affected buccal bone repair.

Our findings are in line with previous research [32], indicating that the absence of vertical incisions in mVIP-CTG enhances flap vascularization and stabilizes the coronal border, hence mitigating flap shrinkage. A potential reason for the protective effect on the buccal bone plate is that the interdental papillae are not raised but maintain their correct location.

Both groups experienced passive, tension-free flap closure, which is optimal for this operation, and the study’s results can be used for surgical flap design. The split thickness technique, which entails positioning mVIP-CTG or XCM within the buccal pouch, maintains the integrity of the periosteal vascularization in the flap.

### Limitations

This clinical trial possesses numerous limitations. The limited sample size (*n* = 20) may diminish statistical power and constrain the generalizability of the findings. Despite the absence of participant attrition during the trial, the initial sample size calculation failed to consider potential dropouts, which may introduce a risk of underpowering in studies with extended follow-up durations. The follow-up duration was restricted to 6 months, potentially inadequate for assessing long-term soft tissue stability, aesthetic results, or bone remodeling. Third, although the mVIP-CTG procedure shows advantageous outcomes, it necessitates graft procurement from the palate, hence prolonging surgical duration and elevating patient morbidity in comparison to readily available options such as XCM. The study was retroactively registered on ClinicalTrials.gov (NCT06808243), with ethics approval secured prior to patient recruitment. This postponed registration diverges from ICMJE recommendations for prospective trial registration and is recognized as a methodological restriction. Subsequent studies will comply with prospective registration protocols to enhance research transparency.

## Conclusion

The current study indicates that employing mVIP-CTG alongside immediate implant placement and bone grafting in the jumping gap leads to a superior enhancement in keratinized tissue thickness compared to XCM, while maintaining buccal cortical bone thickness due to preserved vascularity. While mVIP-CTG demonstrated a statistically significant enhancement in keratinized tissue thickness, its overall superiority in aesthetic outcomes was not significant. Consequently, XCM continues to be a viable and less invasive alternative to CTG in suitable clinical contexts.

### Recommendations

Future research with bigger sample sizes and prolonged follow-up periods is advised to validate and elaborate on the findings of the present study. Longitudinal data would yield significant insights into the stability of peri-implant soft and hard tissue outcomes. Subsequent investigations should examine the biological properties of various soft tissue substitutes, focusing on their integration, degradation kinetics, and impact on tissue regeneration, especially in conjunction with diverse flap designs, such as full-thickness and split-thickness approaches. Standardized techniques for quantifying soft tissue characteristics and evaluating aesthetic outcomes should be implemented to enable comparisons across research. Patient-reported outcome measures (PROMs) should be used to assess the effects of grafting procedures on comfort, satisfaction, and quality of life.

## Data Availability

The datasets generated and/or analyzed during the current study are available from the corresponding author upon reasonable request (Dr. Heba Ahmed Abdelmaged, heba.elsweefy@mu.edu.eg, +20 106 428 8441).

## Abbreviations

XCM: Xenogenic collagen matrix membrane
PES: Pink Esthetic Score
KTW: Keratinized Tissue Width
KTT: Keratinized Tissue thickness
BCT: The buccal cortical thickness
IIP: Immediate implant placement
